# Custom-Technology Single-Photon Avalanche Diode Linear Detector Array for Underwater Depth Imaging

**DOI:** 10.3390/s21144850

**Published:** 2021-07-16

**Authors:** Aurora Maccarone, Giulia Acconcia, Ulrich Steinlehner, Ivan Labanca, Darryl Newborough, Ivan Rech, Gerald S. Buller

**Affiliations:** 1 Institute of Photonics and Quantum Sciences, School of Engineering and Physical Sciences, Heriot-Watt University, Edinburgh EH14 4AS, UK; uks2@hw.ac.uk (U.S.); g.s.buller@hw.ac.uk (G.S.B.); 2 Department of Electronics, Information and Bioengineering, Politecnico di Milano, 20133 Milano, Italy; giulia.acconcia@polimi.it (G.A.); ivangiuseppe.labanca@polimi.it (I.L.); ivan.rech@polimi.it (I.R.); 3 Sonardyne International Ltd., Blackbushe Business Park, Yateley, Hampshire GU46 6GD, UK; darryl.newborough@sonardyne.com

**Keywords:** single-photon, TCSPC, underwater imaging, SPAD, custom technology, 3D imaging, LIDAR

## Abstract

We present an optical depth imaging system suitable for highly scattering underwater environments. The system used the time-correlated single-photon counting (TCSPC) technique and the time-of-flight approach to obtain depth profiles. The single-photon detection was provided by a linear array of single-photon avalanche diode (SPAD) detectors fabricated in a customized silicon fabrication technology for optimized efficiency, dark count rate, and jitter performance. The bi-static transceiver comprised a pulsed laser diode source with central wavelength 670 nm, a linear array of 16 × 1 Si-SPAD detectors, with a dedicated TCSPC acquisition module. Cylindrical lenses were used to collect the light scattered by the target and image it onto the sensor. These laboratory-based experiments demonstrated single-photon depth imaging at a range of 1.65 m in highly scattering conditions, equivalent up to 8.3 attenuation lengths between the system and the target, using average optical powers of up to 15 mW. The depth and spatial resolution of this sensor were investigated in different scattering conditions.

## 1. Introduction

Active optical depth imaging in scattering underwater environments finds application in several fields, including archeology [1,2], autonomous manipulation [3], inspection, and monitoring [4].

There are several methods to obtain three-dimensional optical images, with triangulation and time-of-flight being commonly used. Triangulation is based on the geometrical relation between the separated source and receiver and the target, used to obtain a depth estimation [5]. Direct time-of-flight approaches measure the return time of optical pulses from transceiver to target, to provide an estimate of depth [6]. These approaches can be used in conjunction with different optical scanning techniques in order to obtain a three-dimensional image of the full target area. For example, one of the most used scanning techniques is based on a synchronous scan approach, which scans the target by using a highly collimated laser source and synchronously collects the scattered signal with a receiver having a narrow field of view [7]. When this scanning technique is used with the triangulation approach, the advantage is improved depth resolution when compared with time-of-flight sensors at short ranges (less than 3 m) [8]; however, the main challenge is the calibration process to obtain the correct geometrical parameters of the system [9,10]. The use of the time-of-flight approach overcomes much of the need for a calibration process, however, the depth resolution depends on the temporal resolution of the timing measurement in addition to accurate estimations of the refractive index of the transmission medium.

Over the years, several imaging systems were developed by using the synchronous scanning approach, and most of them use a laser line scanner (LLS) configuration. For example, the system described by Palomer et al. [10] is a LLS based on triangulation, and uses a 50 mW average power green laser in conjunction with a CCD camera. The scanning was performed by using a galvanometer mirror mounted below an underwater vehicle. Three-dimensional imaging was demonstrated at various distances between 0.5–5 m, depending on water attenuation, as a visual aid for underwater manipulation [11] and simultaneous localization and mapping (SLAM) [12]. Another example of LLS is reported in [8,13], which is based on the time-of-flight approach. The receiver was a silicon photodiode and the source was a pulsed laser with central wavelength of 532 nm. The scans were performed by two galvanometer mirrors that scanned the laser line over two directions. This approach demonstrated three-dimensional imaging over a distance in the range 2–40 m, depending on the local attenuation levels in water. Typically, these systems require a scanning mechanism to scan the laser line across the field of view. This approach has been modified to exploit the motion of underwater vehicles to remove the need for scanning optics in the system [14,15,16], which greatly simplifies the complexity of the optical configuration of the transceiver system.

Recently, underwater three-dimensional imaging has been demonstrated by using the time-of-flight approach in conjunction with the time-correlated single-photon counting (TCSPC) technique [17]. The TCSPC technique is a statistical sampling approach based on repeated measurement of the timing difference between the outgoing laser pulse and a target return photon event triggered by a single-photon detector [18]. The target is illuminated by a high-repetition rate pulsed laser source, and the scattered signal that arrives back at the detector is very weak, meaning that the probability of detection from a single laser pulse is low (typically <<5%). Under these conditions, the timing distribution can provide an accurate representation of the time-of-flight of the photon return. Typically, a single-photon lidar system will have a timing jitter of a few hundred picoseconds or lower, however the depth (or timing) resolution will improve further as the number of return photons is increased allowing depth resolution which can be considerably better than that predicted from the timing jitter alone as described by Pellegrini et al. [19]. This leads to a trade-off between measurement time and depth resolution. With an appropriate photon return, depth resolution in the sub-millimeter range is possible, allowing for better distinction between closely spaced surfaces, such as depth profiling of camouflaged targets [20] or objects partially hidden by foliage [21]. The high optical sensitivity and excellent depth resolution make this technique particularly suitable for imaging in challenging conditions, such as depth imaging through smoke or fog [22,23], and kilometer range depth imaging [24,25].

The TCSPC approach has been adapted for use in highly scattering underwater environments, and depth profiling was achieved using sub-milliwatt average optical powers with scattering levels up to 9.2 attenuation lengths (AL) between transceiver and target [17,26], i.e., 18.4 attenuation lengths round-trip (one attenuation length being the distance the light travels before its intensity is reduced to 1/e of its initial value). The demonstration of this technique in underwater environments was first performed by an optically scanning monostatic transceiver fiber-coupled to an individual single-photon avalanche diode (SPAD) detector. The monostatic transceiver raster scanned the laser illumination across the target area using two galvanometer mirrors, with the return signal being transferred to the SPAD along a common optical axis with the outgoing illumination. This allowed a narrow field of view of approximately 0.5 mrad, meaning that forward scattering events could be effectively spatially filtered, as they are unlikely to be collected in the narrow optical field of view of the receiver optics. With picosecond detection resolution, back-scattered events can be distinguished from the return signal in the time, or depth, domain. Use of single-pixel detector approach meant that a high spatial resolution of less than 60 μrad was achieved in scattering waters up to 8 AL between the system and the target. However, this was at the expense of long acquisition times to acquire the timing information over the whole target area. In order to avoid time-consuming optical scanning and achieve shorter acquisition times, later work focused on the use of SPAD detector arrays, initially using a 192 × 128 pixel SPAD detector array [27]. This SPAD detector array was fabricated in complementary metal-oxide semiconductor (CMOS) technology, with timing electronics integrated into each pixel [28]. Taking into account the fill-factor, the overall detection efficiency of each pixel was 0.9% at λ = 670 nm. The average dark count rate of the array was 28 cps per pixel, and the instrumental response of the system was approximately 350 ps. Depth imaging in scattering environments equivalent to 6.7 AL between transceiver and target were achieved, using average optical powers of up to 8 mW. Although this result is very promising, the optical configuration meant it was not possible to achieve imaging at the levels of scatter possible with the single-pixel system, since the larger field of view of the SPAD detector array sensor meant that more scattering events were detected than in the case of the single-pixel detector geometry. This led to shorter achievable ranges and reduced spatial resolution when compared with the earlier single-pixel detector configuration. Critically, the SPAD detector array approach did, however, allow simultaneous full-field depth measurements at a large pixel format in data acquisition times as short as 10’s μs.

Typically, CMOS SPAD detector arrays give the advantage of a high degree of parallelization [29], but the constraints of the fabrication process mean that the performance of the SPAD detectors cannot be optimized to the level possible with a customized SPAD fabrication process [30], which can allow the tailoring of detector characteristics. When compared with CMOS SPAD detector arrays, SPAD detectors fabricated in custom technology present several advantages, such as increased single-photon detection efficiency (above 30% at λ = 670 nm [31]), and lower timing jitter (of the order of 35 ps [32]). Additionally, higher maximum count rates and lower dark count rates can also be achieved when custom fabrication processes are used. For example, a count rate of 120 Mcps and dark count rate of 9 cps at the temperature T = −20 °C has been demonstrated by Ceccarelli et al. [33]. It is important to note that when SPAD detectors fabricated in custom technology are used for time-correlated measurements, external front end and timing electronics are needed [34,35,36,37], potentially limiting the number of pixels. However, at the same time, a high number of pixels means a high count rate of the module; under such conditions, readout times become significant and may limit the speed of a TCSPC measurement [38].

In this paper, we investigate a Si-SPAD detector array fabricated in custom technology optimized for underwater three-dimensional imaging. The hardware consists of two main parts: a detection head and a TCSPC module. The detection module is based on the architecture reported in [39,40], with a new firmware specifically developed to meet the requirements of underwater depth imaging. The TCSPC module asynchronously time-tagged the events detected by the detection head, providing the timing information needed to build the depth profile. In this work, the system was kept stationary and the target was shifted in water with a motorized stage to perform the scan. This is equivalent to a system integrated in an underwater vehicle and driving the scan by using the direction of travel of the vehicle, avoiding the use of scanning optical components. The motivation for this work is to exploit the high data rate possible with SPAD detector arrays fabricated in custom technology, while reducing the optical field of view to a value much lower than required with a rectangular SPAD array, as demonstrated previously [27]. Coupled with the lack of optical scanning, this can mean a high performance depth imaging system capable of operation in high scattering levels, with the benefit of relatively low size, weight, and power requirements.

## 2. Materials and Methods

### 2.1. Detection Head

The detection head is the core of the detection system. First of all, it hosts a 16 × 1 array of custom silicon SPADs [41], featuring a photon detection efficiency (PDE) as high as 28% at λ = 670 nm and an average dark count rate (DCR) of 924 cps. The diameter of each SPAD is 50 µm and the pitch is 250 µm. Moreover, a 16 × 1 array of fully-integrated active quenching circuits (AQCs) is placed close to the SPAD array to minimize parasitics, thus ensuring a fast sense of the avalanche current, promptly followed by an active quenching phase. In this way, it is possible to minimize the amount of charge flowing through the SPAD during an avalanche event, an aspect that is crucial to minimize the afterpulsing probability [42]. Both the SPAD and the AQC array are placed in a sealed chamber where a dry atmosphere is created by purging nitrogen through the frontal valves. In this way, it is possible to cool down the detectors to achieve significant reduction in dark count rates [41], while avoiding any potential issue due to moisture. A closed loop temperature control based on a thermo-electric cooler (TEC) allowed us to cool the SPADs down to 0 °C. Finally, the detection head generates the timing signals which are fed to the TCSPC module via a high-speed and low-crosstalk differential Samtec cable.

### 2.2. TCSPC Module

The timing signals extracted from the SPAD array require a time measurement circuit able to convert such information into a digital word for processing. To this aim, our TCSPC module is based on four fully-integrated 4-channel arrays of time to amplitude converters (TACs), featuring the structure reported in [43], followed by two 8-channel commercial 14-bit ADCs (AD9252 from Analog Devices). Overall, the TCSPC module receives the Start signals from the detection head, each one marking the detection of a photon, and the Stop signal from the laser. The Start-Stop time interval is converted into a digital word by the TACs followed by the ADCs, and then sent toward an external PC via USB3.0. In particular, in this module we developed a new firmware to implement the time-tagging data transmission method, meaning that each event is described by two strings: a macro time, i.e., the coarse time elapsed by the start of the measurement which is obtained by counting the number of laser periods, and the micro time, i.e., the precise position of the detection event within the laser period which is measured by the TAC. Overall, the detection system features a timing bin-width of 1.6 ps, a timing jitter of ~60 ps full width at half maximum (FWHM), and a differential non-linearity (DNL) lower than 4% peak to peak of the LSB. Each channel provides a conversion rate up to 4 Mcounts/s, thus achieving an overall rate as high as 64 Mcounts/s.

### 2.3. Experimental Setup

**Figure 1:**
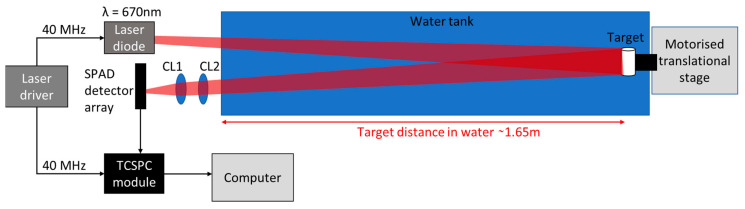
Schematic of the experimental setup. The system comprised a pulsed laser diode source with central wavelength 670 nm, a SPAD detector linear array, and dedicated TCSPC electronics. The scan was performed by moving the target over the vertical direction with a motorized translational stage, and the light scattered by the target was collected by using two cylindrical lenses (CL1 and CL2).

Figure 1 shows the schematic of the experimental setup. The illumination source used was a pulsed semiconductor laser diode (PicoQuant LDH-P-C-670M, PicoQuant GmbH, Berlin, Germany) with central wavelength λ = 670 nm, a repetition rate equal to 40 MHz, and a pulse-width of approximately 120 ps that dominated in the overall temporal response of the system. The operational wavelength was selected to correspond with the spectral maximum transmittance in highly scattering environments (>5 AL), as demonstrated previously [17]. The laser was highly diverging in one axis, which was orientated along the horizontal axis at the target position. The target was placed in a 1750 mm long, 250 mm wide, 250 mm high water tank. The distance of the target in water was approximately 1.65 m. During a depth profile measurement, the target was moved along the vertical direction using a motorized translational stage. The speed of the target was 10 mm/s and the distance travelled by the target was 100 mm, which meant that all the scans shown in this initial demonstration were performed by using an overall acquisition time equal to 10 s.

Two cylindrical lenses (CL1 and CL2) were used to collect the light scattered by the target and focus the illuminated area efficiently onto the linear SPAD detector array. The lenses had a clear aperture of approximately 23 mm, and focal lengths f_1_ = 75 mm and f_2_ = 150 mm. These focal lengths were chosen in order to match the laser line dimensions (35 mm × 2 mm at the target position) to the size of the linear SPAD detector array.

Before each depth profile measurement, the attenuation of the environment was measured by using the method and setup described in [27]. This method allowed us to collimate the light from the laser source and measure the average optical power at two positions, *P*_1_ and *P*_2_, separated by a distance *d* = 0.5 m in water. From the Beer–Lambert law [44], the transmittance of the water over half a meter was then calculated as

(1) $$T=\frac{P_{2}}{P_{1}}=e^{-\alpha d}$$

where *α* is the attenuation coefficient of the environment and *αd* is the number of attenuation lengths.

During a depth profile measurement, the TCSPC module asynchronously time-tagged the detected events and the synchronization signal for the entire duration of the scan. This meant that the vertical resolution of the image (i.e., the number of lines per scan) was set by the user during the analysis of the data, and shorter acquisition times per line were investigated without the need of repeating the measurement.

For each pixel, a histogram was built with the desired acquisition time and a temporal resolution of approximately 1.6 ps, which was equivalent to 0.18 mm depth in water. A pixel-wise cross-correlation *X* was performed between the histogram recorded *H* and the normalized instrumental response *C* recorded for each channel, as

(2) $$X_{i}=\overset{t}{\underset{i=1}{\sum }}H_{i+k}\times C_{i},\;\;\;\;\;\;\;\;\;\;\;\;\;\;\;k\in \left[-t,t\right]$$

where *t* is the number of timing bins used for the calculation. The maximum of the cross-correlation for each pixel was used as an estimate of the time-of-flight and provided the relative depth with respect to the reference. The intensity for each pixel was estimated by counting the number of events in a time interval of 150 bins width centered on the maximum value determined by the cross-correlation.

**Figure 2:**
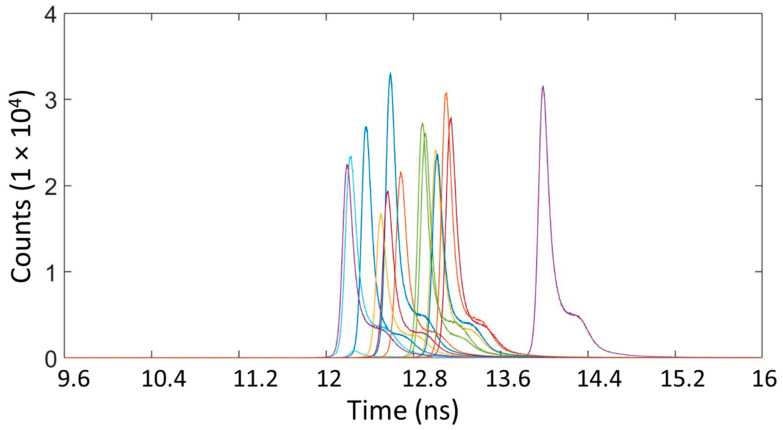
Instrumental response of the 16 detectors recorded in unfiltered tap water using an acquisition time of approximately 40 s.

Figure 2 shows the instrumental timing response of the system for each of the detectors. This instrumental response is the timing response for the entire imaging system, and is the convolution of the temporal responses of the laser system, the detectors and the timing electronics. The instrumental response measurement was performed in unfiltered tap water, using a Lambertian reference flat target with nominal reflectance equal to 99% (Spectralon Diffuse Reflectance Target, Labsphere, North Sutton, NH, USA). The average optical power used was 27 nW, and the acquisition time was approximately 40 s. The repetition rate of the synchronization signal was 40 MHz, corresponding to a period of 25 ns in the histogram. The instrumental response was measured over the full period; however, Figure 2 displays a reduced timing interval of approximately 6.5 ns in order to illustrate the instrumental responses with greater clarity. The relative delays in the peak returns from the flat reference target in the instrumental response are fixed delays between the detectors, which are due to small differences in the output voltages of the integrated pick-up circuit of each detector. Hence, it is vital to perform the cross-correlation using the raw data and instrumental timing response from each individual detector, rather than an average timing response over the whole array. In addition, the histogram in Figure 2 shows a shoulder on the right side of each peak, which originated from the pulsed laser diode source used in the experiments. Therefore, to maintain an identical temporal profile of the laser pulse in all the measurements, the driving current of the laser was chosen in order to maximize the overall energy in the pulse while maintaining an individual peak in the temporal profile. The use of higher driving currents would increasingly cause a second peak to become more dominant, which would eventually affect the timing, and depth, resolution possible. This meant a maximum average optical power of approximately 15 mW was used for the scans in highly scattering environments (>6 AL). At lower scattering levels, the average optical power was reduced with the use of ND filters in order to avoid saturation of the detector.

The pixel-wise cross-correlation approach gives an accurate estimation of the time-of-flight of the detected photons when the measurement is performed in favorable conditions which typically result in a high photon return in the histogram, as in the case of absence of scattering in water or long acquisition time per pixel. However, shorter acquisition times or high level of scattering will reduce the signal to noise ratio, which can eventually cause an incorrect estimation of the depth [19].

Several methods have been developed to improve the depth estimation in the presence of scattering. For example, the cross-correlation approach can be performed by selecting the timing bins of the histogram where the photon return from the target is expected. This approach does not include the backscattering from shorter depths than the target position in the calculation, which can improve the depth estimation of the area of interest from a cross-correlation approach. In the results shown in this paper, the cross-correlation approach was performed by selecting in each channel a timing interval of 1000 bins in the overall 16,384 timing bins recorded in the original histogram. However, this approach assumes that the approximate location of the target is known in order to select an appropriate timing bin position. If the target position is not known, advanced computational imaging approaches can be used to analyze the entire histogram and to reconstruct complex scenarios with very low numbers of returned photons [45].

The main parameters of the experiment are summarized in Table 1.

## 3. Results

**Figure 3:**
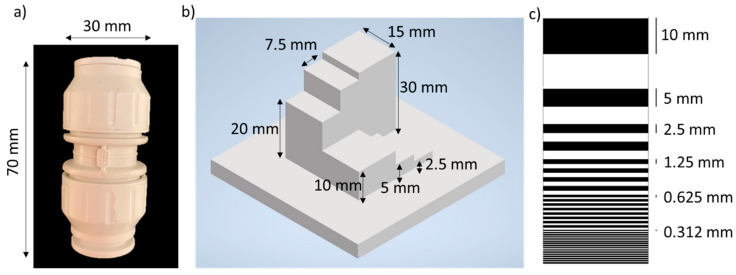
Targets used in the experiments. (**a**) Photograph of a plastic pipe connection. (**b**) Schematic of target used to investigate the depth resolution of the system in scattering environments. (**c**) Schematic of target used to investigate spatial resolution of the system in a number of scattering conditions.

The target used in the depth and intensity measurements is shown in Figure 3a. This target was a commercial plastic fitting pipe connector, 70 mm long and with external diameter equal to 30 mm. The depth resolution of the system was investigated in different scattering environments using the depth target shown schematically in Figure 3b. The depth target was designed and 3D printed in order to have depth features in the range from 2.5 to 30 mm. The third target shown in Figure 3c is a flat black and white target printed on adhesive vinyl. The target consisted of a set of horizontal lines with different thicknesses, varying from 0.312 to 10 mm, and it was used to study the spatial resolution of the system when a user defined number of lines was used to reconstruct the image in unfiltered tap water and scattering environments.

### 3.1. Depth Imaging in Non-Scattering Water

The performance of the system was initially investigated in unfiltered tap water using the plastic pipe target shown in Figure 3a. In this environment, the attenuation between the system and the target was relatively low, equivalent to 1.2 AL at λ = 670 nm. For this measurement, the average optical power entering the water tank was 9 μW. The overall acquisition time of the scan was 10 s, and the data were analyzed by selecting a vertical resolution of 1000 lines, which was equivalent to 10 ms acquisition time per line. Additionally, shorter acquisition times were extracted by the recorded histograms and analyzed with the pixel-wise cross-correlation approach.

**Figure 4:**
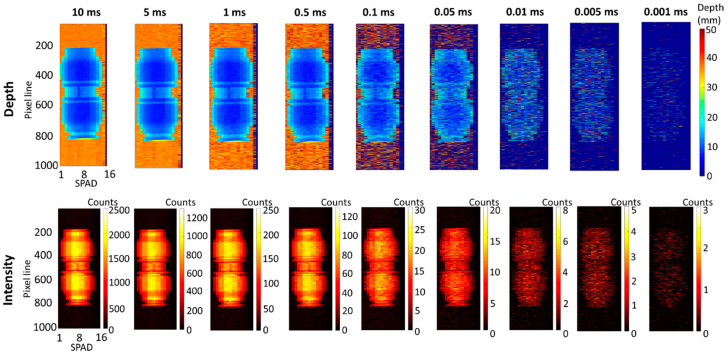
Depth (**top line**) and intensity (**bottom line**) profiles of the plastic pipe target in unfiltered tap water (1.2 AL). The acquisition time per line was varied by software from 10 ms to 1 μs, and the average optical power was equivalent to 9 μW. Under these conditions, the average target return rate for this measurement was approximately 1231 counts per pixel per 10 ms acquisition time, obtained performing the average over 8 SPAD detectors in the central part of the target.

The depth and intensity profiles are shown in Figure 4 in the top line and bottom line, respectively. The results are shown for decreasing acquisition times from 10 ms to 1 μs. The depth and intensity profiles at the longest acquisition time of 10 ms show the sub-millimeter depth resolution achievable by the system. As the acquisition time per line is decreased, the resolution of the image decreases accordingly. However, the target can be reconstructed at the acquisition time per line of 5 μs, and still be detected at the acquisition time per line of 1 μs.

### 3.2. Depth Imaging in Scattering Underwater Environments

The second experiment investigated the depth imaging capabilities of the system in a number of scattering environments. In order to increase the level of scattering of the water, an antacid medicine (Maalox suspension) was used as scattering agent [46].

The plastic pipe target was kept at the distance of 1.65 m in water; however, by adding the scattering agent to unfiltered tap water, the number of attenuation lengths between the transceiver and the target increased as described by Equation (1).

The scans were acquired with an overall acquisition time of 10 s and the data were analyzed choosing a vertical resolution of 1000 lines. Hence, the histograms were built using an acquisition time per line of 10 ms, and then analyzed by performing the pixel-wise cross-correlation approach to reconstruct the depth and intensity profiles of the target.

**Figure 5:**
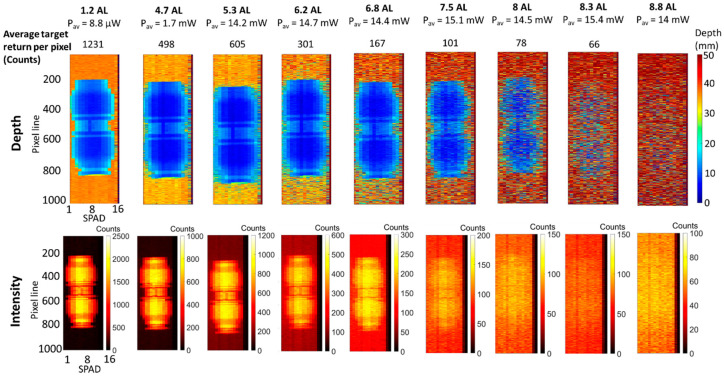
Depth (**top line**) and intensity (**bottom line**) profiles of the plastic pipe target in unfiltered tap water and several concentrations of scattering agent. The data were analyzed with the pixel-wise cross-correlation approach, the acquisition time per line was set to 10 ms, and the average optical power was adjusted from 9 μW to 15.4 mW, depending on the level of scattering in water. The average target return for each scan was obtained by averaging the count rate of the 8 SPAD detectors in the central part of the target over the 10 ms acquisition time.

The main results are shown in Figure 5. The first column shows the case of unfiltered tap water, which was equivalent to an attenuation of 1.2 AL between the transceiver and the target. The next columns show the increasing level of scattering in water up to 8.8 AL. As the level of scattering is increased, the depth resolution of the depth profile decreases because of the lower photon return from the target and the higher level of background. With additional attenuation, the backscatter increases the background count level as well as reducing the signal strength, leading to a deterioration in the quality of the reconstructed image. However, the results show that the target can be reconstructed with the cross-correlation approach up to 8 AL. At higher levels of scattering, the target can be detected up to 8.3 AL but then the target cannot be distinguished from the background, preventing the depth estimation. The average optical power of each scan was adjusted using ND filters depending on the level of scattering, to optimize the return from the target without saturating the SPAD detector array. The average target return in each environment indicates the average return from the target calculated by adding the events in a timing interval of 150 bins width centered on the timing bin with the maximum value of the cross-correlation. In addition, the contribution of the background was subtracted by the return peak, and it was calculated over 150 bins next to the return peak from the target. Then, the average was performed over the 8 SPAD detectors in the central part of the target only.

At the same time, the intensity map was reconstructed by counting the number of events around the maximum of the cross-correlation. The profiles in different scattering environments are shown in the bottom line of Figure 5, where it is possible to discern the target up to 6.8 AL but then the target gradually disappears as the level of scattering is further increased.

Table 2 shows the average target return per pixel without the background contribution, and the signal-to-background ratio (SBR) defined as the ratio between number of counts in highest bin in the peak and the average background per bin. The values in the table were calculated and averaged over 10 ms acquisition time for the central 8 pixels of the detector array. When the SBR >2, there is no loss of information from the target and the image is fully reconstructed. At increased level of scattering (>7 AL), the SBR decreases because of the attenuation of the signal from the target and the higher background, meaning that for SBR <1.2 the target cannot be detected with the cross-correlation approach. However, depth information retrieval can be achieved with bespoke image processing techniques that exploit spatial correlation between adjacent pixels. This was shown in previous work in highly scattering environments [47]. In addition, it is important to note that the main contribution to the background level is due to scattering events, with a negligible contribution from detector dark counts. The average detector dark count is 0.09 counts over 150 bins in a 10 ms acquisition time, meaning that under these conditions the level of scattered background events is approximately 100 times the contribution from detector dark events alone.

### 3.3. Depth and Spatial Resolution

**Figure 6:**
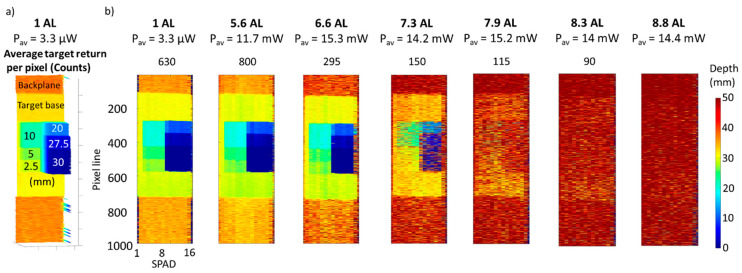
(**a**) Depth profile of the depth target in unfiltered tap water, equivalent to 1 AL between the system and the target. The figure shows the height of each block in mm with respect to the base of the target. (**b**) Depth profiles of the depth resolution target in unfiltered tap water and several concentrations of scattering agent. The data were analyzed with the pixel-wise cross-correlation approach, the acquisition time per line was set to 10 ms, and the average optical power was adjusted from 3.3 μW to 15.3 mW, depending on the level of scattering in water. The average target return per pixel was calculated over the entire array for each scan, from line 200 to 600, using an acquisition time of 10 ms.

In order to investigate the depth resolution achievable by the system in different scattering environments, several scans of the depth target (Figure 3b) were performed by varying the level of scattering in water. Figure 6 shows the depth profiles of the depth target in a number of scattering environments. The average optical power of the measurements was varied in the range from 3.3 μW to 15 mW, depending on the level of scattering of the water. The data were analyzed with the cross-correlation approach by using 1000 lines and an acquisition time per line of 10 ms. The profile Figure 6a shows the height of each block of the target with respect to the base of the target as seen from the system. In ideal conditions such as long acquisition times and absence of scattering, the depth resolution of the profile is limited only by the timing resolution of the system and the number of counts detected. In these experiments, the timing histogram bin width used was 1.6 ps, which allows to achieve sub-millimeter depth resolution. However, the aim of this study is to quantify the degradation of the depth resolution with scattering, and hence the target was designed and printed in order to have depth features of the order of few millimeters (≥2.5 mm).

The results in Figure 6 show that depth resolution of better than 2.5 mm is maintained up to 6.6 AL and it starts degrading at 7.3 AL. Depth features of the order of 1 cm can still be retrieved at 7.9 AL, but at higher levels of scattering the depth structure of the target cannot be discerned, and the target cannot be detected at all at attenuation levels above 8.3 AL.

**Figure 7:**
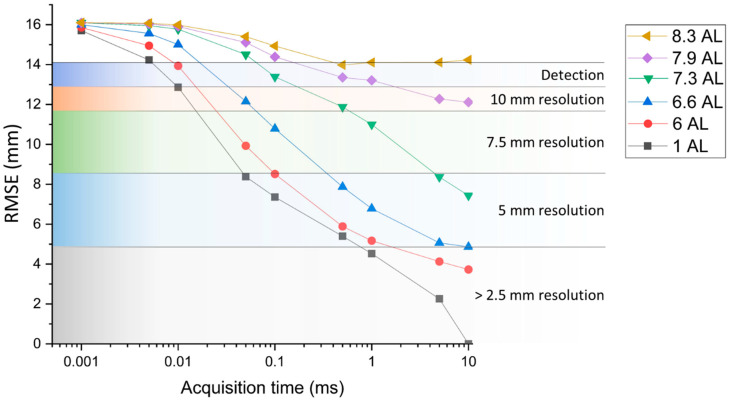
RMSE versus acquisition time for several underwater scattering environments. The colored regions of the graph highlight the achievable depth resolution for each RMSE range value.

In order to quantify the degradation of the depth resolution with scattering, the depth estimation in each environment was compared with the measurement performed in unfiltered tap water by using the range root mean square error, defined as [48]:

(3) $$RMSE\;=\;\sqrt{\overset{N}{\underset{n\;=\;1}{\sum }}{\left(d_{n}-x_{n}\right)}^{2}}\;$$

where *x* is the depth estimated in the profiles, and *d* is the depth estimated in the reference scan, which was the case of unfiltered tap water and acquisition time per line equal to 10 ms. The comparison was performed only over the pixels corresponding to the target area, which means that the backplane was not considered in the calculations and the number of pixels *n* in Equation (3) was varied in the target area from line 200 to line 600, i.e., *N* = 6400. In addition, shorter acquisition times were investigated for each environment and compared with the chosen ground truth. The RMSEs calculated for each set of data are shown in Figure 7, along with the achievable depth resolution obtained by inspecting the depth profiles retrieved with the cross-correlation approach. The graph shows that the depth resolution of the image in unfiltered tap water starts to deteriorate at the acquisition time per line of 0.5 ms, and centimeter resolution is achievable at 0.01 ms. At shorter acquisition times, the target can be resolved using acquisition times per line up to 0.005 ms, and cannot be detected beyond this limit. In the case of the pipe connector target, detection was possible at shorter acquisition times due to the higher reflectivity of the target.

**Figure 8:**
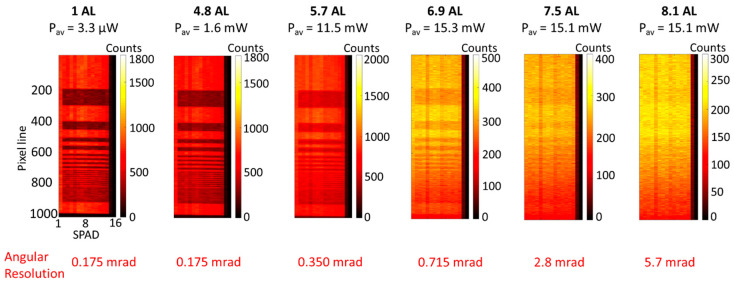
Intensity profiles of the variable lines resolution target (as shown in Figure 3c) in unfiltered tap water and several concentrations of scattering agent. The acquisition time per line was set to 10 ms, and the average optical power was adjusted from 3.3 to 15.3 mW, depending on the level of scattering in water.

The spatial resolution of the system was investigated performing the scan of the variable lines resolution target (Figure 3c) in several scattering environments. The data were analyzed with the cross-correlation approach, choosing 1000 lines and an acquisition time per line of 10 ms, which are the same parameters used in the previous sections. The intensity maps of the target are shown in Figure 8, and the average optical power of the scans were adjusted according to the level of scattering of the water, varying in the range from 3.3 μW to 15 mW. The lines of thickness equal to 0.312 mm can be resolved in attenuation levels up to 4.8 AL, which is equivalent to an angular resolution of 175 μrad. However, due to the forward scattering events detected, the achievable angular resolution increases to 5.7 mrad at 8.1 AL, and at higher attenuation levels it is not possible to resolve the target in the intensity map.

## 4. Conclusions

This work demonstrates underwater optical imaging based on the time-of-flight approach and the TCSPC technique using a SPAD detector linear array built in custom technology. The detection module comprised a 16 × 1 array of custom fabricated silicon SPADs with high detection efficiency and low timing jitter. The array was connected to a dedicated TCSPC module that provided the timing information with timing bins of only 1.6 ps, with a maximum overall count rate of 64 Mcps.

Laboratory based experiments were performed over a distance of 1.65 m in water, with a non-scanning optical configuration and the system asynchronously streaming time-tagged events from a moving target. This configuration was chosen in order to simplify the optical configuration of the system, which avoided the need for scanning optics. Additionally, it simulated a configuration where the system is integrated on an underwater vehicle, driving the scan of the target area with the direction of travel. A number of acquisition times per line were investigated to study the shortest acquisition times that allowed the reconstruction of depth profiles of the moving target, demonstrating depth profiles at acquisition times as short as 1 μs per line in clear water. In addition, depth and intensity profiles of targets were obtained in a variety of underwater scattering environments, which demonstrated depth reconstruction in scattering conditions equivalent up to 8.3 AL between the transceiver and the target. These experiments were performed in controlled laboratory conditions, with an intentionally low ambient background light level. In an outdoor underwater environment, there will be a contribution from the solar background which will add uncorrelated background events to the histogram. However, the optical setup of the transceiver can be modified in order to include optical bandpass filters to reduce the detection of ambient light in the receive channel, as demonstrated in previous work in free space single-photon lidar performed in high ambient light levels in the near and short-wave infrared [22,23,24,25,49].

Depth and spatial resolutions were investigated using reference targets. The depth profiles demonstrated that millimeter depth resolution was preserved in highly scattering environments up to 6.6 AL between transceiver and target, whereas centimeter depth resolution can be obtained up to 8 AL. At higher levels of scattering, detection is possible up to 8.3 AL but the profile of the target cannot be reconstructed beyond this limit. At the same time, the spatial resolution was investigated in several scattering environments, showing that the angular resolution was equal to 175 μrad up to 4.8 AL, and gradually degraded to 5.7 mrad at 8 AL. However, it is important to note that the results obtained in this work were analyzed by using the pixel-wise cross-correlation approach and did not use bespoke image processing techniques which can greatly improve the depth and spatial resolution of the reconstructed image and allow image reconstruction at lower levels of return photon [22,45,47,50,51].

When compared with the single pixel scanning transceiver system based on the TCSPC technique [17], the use of a linear array of Si-SPAD detectors built in custom technology gives the advantage of achieving faster overall acquisition times, but the use of a wider FOV means that more scattering events are detected limiting the depth reconstruction up to 8.3 AL, against the 9.2 AL achievable with the single-pixel approach [47]. Faster acquisition times were achieved with an optical system based on a CMOS SPAD detector array [27], which allowed depth profiling up to 6.7 AL using synchronous data acquisition at 500 Hz. This system gave the advantage of a large pixel format (192 × 128), but the CMOS SPAD detector array fabrication process meant a lower performance of each SPAD detector, which in conjunction with the larger FOV required by the geometry of the array limited the depth profiling capabilities in highly scattering underwater environments. The results presented in this work show that SPAD linear detector arrays have the potential for the detection of low scattering return due to the comparatively narrow optical field of view, while maintaining a high data rate. These linear detector array configurations can act as an ideal compromise between the narrow field of view and slow acquisition of single-pixel systems, and the high data rate of rectangular detector arrays which have reduced contrast and spatial resolution due to greatly increased detection of excess scattering events caused by their necessarily large optical field of view. Such linear SPAD detector arrays have the advantage of more easily allowing the use of custom fabrication technology, permitting optimized single-photon performance. The use of linear detector arrays can also remove any requirement for bulky scanning optics when using the forward movement of the underwater vehicle.

Future work will investigate the possibility of developing larger linear arrays of custom Si-SPAD detectors optimized for this application. Further improvements will include the investigation of a fast gating signal to switch the detectors on for a short temporal window to reduce the overall detection of backscatter events, and the development of bespoke image processing algorithms to retrieve the depth and intensity profile in highly scattering conditions.

## Figures and Tables

**Table 1:** Main parameters of the experiments.

Parameter	Value
Laser source	Laser diode (PicoQuant, Germany)
Illumination wavelength	670 nm
Repetition rate	40 MHz
Average optical power	3.3 μW–15 mW (power varied with ND filters at the output of the laser head)
Stand-off distance in water	1.65 m
Illumination beam at target	35 × 2 mm
Detector	16 × 1 silicon SPAD detector fabricated in custom technology
Timing module	Dedicated TCSPC electronics
Histogram bin width	1.6 ps in average
Instrumental response at FWHM (includes laser, detectors, and timing electronics)	121 ps on average across the array
Optical field of view (full-angle)	18.9 × 0.4 mrad
Dark counts	923.8 cps
Overall background light in clear water (incl. dark counts)	1354.6 cps
Scan area	33 × 100 mm
Target speed	10 mm/s

**Table 2:** Average target return per pixel and signal-to-background ratio (SBR) for the scans shown in Figure 5. The averages were calculated by considering the central 8 SPAD detectors for those pixels coincident with the target.

Attenuation Lengths	Average Optical Power (mW) from Source	Average Target Return Per Pixel in 10 ms Acquisition	SBR
1.2	0.009	1231	6.8
4.7	1.7	498	6.0
5.3	14.2	605	4.3
6.2	14.7	301	3.4
6.8	14.4	167	2.5
7.5	15.1	101	1.9
8	14.5	78	1.5
8.3	15.4	66	1.2

## Data Availability

The data presented in this paper can be found at DOI: 10.17861/27b3b0f1-b696-4795-9110-0ae462338994.
